# Breakpoint Planning Method for Rice Multibreak Milling

**DOI:** 10.3390/foods12091864

**Published:** 2023-04-30

**Authors:** Yawen Xiao, Fuguo Jia, Xiangyi Meng, Yanlong Han

**Affiliations:** 1 School of Mechanical Engineering, Yangzhou University, Yangzhou 225127, China; 2 Jiangsu Engineering Center for Modern Agricultural Machinery and Agronomy Technology, Yangzhou 225127, China; 3 College of Engineering, Northeast Agricultural University, Harbin 150030, China; 4 College of Mechanical Engineering, Jiangsu University of Science and Technology, Zhenjiang 212100, China

**Keywords:** breakpoints planning, multibreak milling, hierarchical cluster analysis, principal component analysis, generalized regression neural network

## Abstract

Excessive milling of rice kernels will result in nutrient loss and grain waste. To avoid grain waste, multibreak milling systems have been widely used in large-scale commercial rice mills. However, there is still no reasonable breakpoint planning method to guide the multibreak milling process. To construct a reasonable multibreak milling system, in this research, taking rice milling, a typical heterogeneous cereal-kernel milling process, as an example, the multivariate analysis method was used to comprehensively analyze the characteristic changes of milled rice during the whole milling process. A breakpoint planning method was established, including planning the number of breakpoints, determining the degree of milling or milling time corresponding to each breakpoint, and estimating the actual breakpoint to which the milled rice belongs. The verification results showed the rationality and high accuracy of the planning method. The presented work will help operators to plan the multibreak milling system of rice efficiently and objectively so as to significantly improve the commercial value of milled rice.

## 1. Introduction

**Figure 1:**
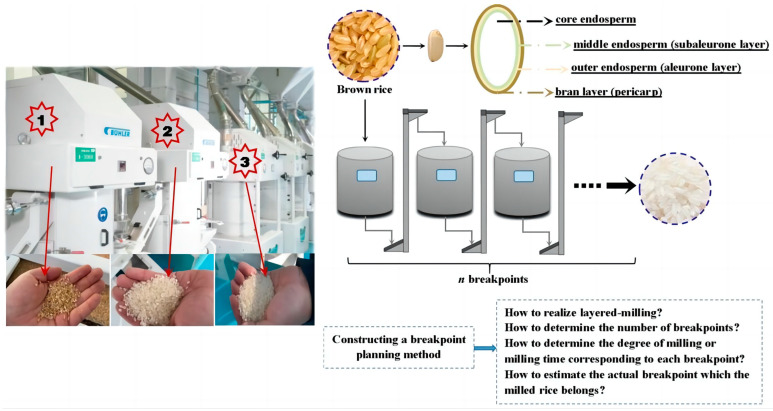
Schematic diagram of multibreak milling system of rice.

A large part of milling operations involves the use of heterogeneous cereal kernels [1,2,3,4,5,6]. In the milling process, the structure and composition of heterogeneous milled materials can be changed markedly, such as the brown rice (BR) whitening process. Whole BR consists of four main fractions (as shown in Figure 1), namely the bran layer (pericarp layer), outer endosperm, middle endosperm (aleurone layers), and core endosperm [7]. Rice milling is a key procedure to remove the pericarp and aleurone layers from the exterior of the BR kernels, and the amount of these fractions removed is referred to as the degree of milling (DOM). In recent years, extensive research has shown that there are the considerable differences in composition and structural strength of each fraction [8,9], thus layered milling based on the microstructure of BR would be quite beneficial for enhancing the quality of the rice milled and meeting the different needs of consumers. A reasonable multibreak milling system is desirable, which enables cereal kernels to reach layered milling. Therefore, multibreak milling systems (a schematic diagram of the multibreak point milling system is shown in Figure 1) have been widely used in large-scale commercial rice mills in recent years [10,11]. However, at present, control on the DOM range for each single break and planning of the number of breakpoints are based primarily on the experience of operators and their proficiency with the processing machinery in practice [12]. As a result, the existing multibreak milling system is not only often unable to achieve layered milling but also brings huge economic losses to large-scale commercial rice mills. For example, due to excessive breakpoints, excess equipment may be idle, and the final product may be overmilled and contain a high breakage rate. On the contrary, too few breakpoints is also unreasonable because it will cause the multibreak milling system to lose the advantage of layered milling or cause the final product to fail to meet the requirements. In addition, how to control the milling time (MT) of each single-breakpoint mill to meet the requirements of layered milling is also an urgent problem to be solved. Consequently, it is of great practical value to explore the changes of milled rice during the whole milling process, and to establish an objective and highly efficient breakpoint planning method based on this.

Recently, with scanning electron microscopy (SEM), many researchers observed the relationship between the bran removal process and DOM in the micro view and gave the range of the DOM corresponding to each fraction of BR [13,14]. No doubt this provides a new technical scheme for the understanding of the bran removal process and control of the rice multibreak milling process. Unfortunately, change in microscopic structure is tough to monitor in rice milling. Physical properties, as the macroscopic reflection of the microscopic structure, can facilitate the assessment of different stages of the bran removal process. For example, Mohapatra and Bal [15] found that there is a transition point of the variation rate on the nonlinear curve of the relationship between color and the DOM. The corresponding DOM at the transition point is in good agreement with the observed result of SEM. However, the physical properties affected by milling are not only numerous but also complex. Extensive research suggests that the physical properties of milled cereal, such as size, shape, density, weight, and friction coefficient, are easy to change significantly in the milling process [16,17,18]. Moreover, in the milling process, changes of the properties are often highly nonlinear, and large volumes of the properties data need to be acquired and processed [19,20,21]. Hence, a scientific method that can effectively analyze the variety law of the milled particulate properties during the milling process is needed. 

Pattern recognition techniques such as principal component analysis (PCA), hierarchical cluster analysis (HCA) and artificial neural networks (ANNs), which allow for visualization of the underlying structure between product properties and products, have been used widely in different fields of engineering [22,23,24]. For example, Barua et al. [25] used PCA and HCA to determine the crucial parameter and ideal processing for altering the rapidly digestible starch fraction from a multitude of processing parameters and conditions. Barros et al. [26] indicated that multivariate analysis allowed the classification of different edible seeds based on nonlinear physical and chemical characteristic parameters such as moisture, total phenolic compounds, and antioxidant activity. Yang et al. [27] achieved a rapid identification of beer species by multivariate analysis of forty volatile compounds. Sharin et al. [28] used multivariate analysis methods including PCA, HCA, and ANN to discriminate between different entomological-origin honey samples based on physicochemical properties and volatile compound characteristics. In overview, a large amount of nonlinear data processed by multivariate analysis can characterize and predict the similarity between samples [29,30,31]. Therefore, it is expected that a reasonable breakpoint planning method can be constructed by using the multivariate analysis method described due to the similarity of many physical properties with the variation of the DOM.

In this study, the method of multivariate analysis was used to comprehensively analyze the characteristic changes of milled rice during the milling process. On this basis, a first attempt of constructing a breakpoint planning method for the multibreak milling system was presented, in order to provide reference for the application of a commercial multibreak milling system.

## 2. Materials and Methods

### 2.1. Experimental Materials

The experimental japonica paddy (Dongnong 429) was supplied by the experiment station of Northeast Agricultural University (Harbin, China). The paddy was hulled by a sheller (THU-35B type, Satake, Tokyo, Japan, dehulling speed: 100–120 kg/h) and BR was obtained after removing unsound kernel, broken kernel, and foreign matter. The initial moisture content of BR was 12.1 ± 0.1% (w.b.), measured using the 105 °C dry oven (DGH-9053A type, Yiheng, Shanghai, China) gravimetric method [32]. Rice samples with similar size, shape, and no cracks were picked. The treated samples were stored in double-sealed polyethylene bags at 5 °C in a refrigerator before milling [15].

### 2.2. Friction-Type Milling Process

**Figure 2:**
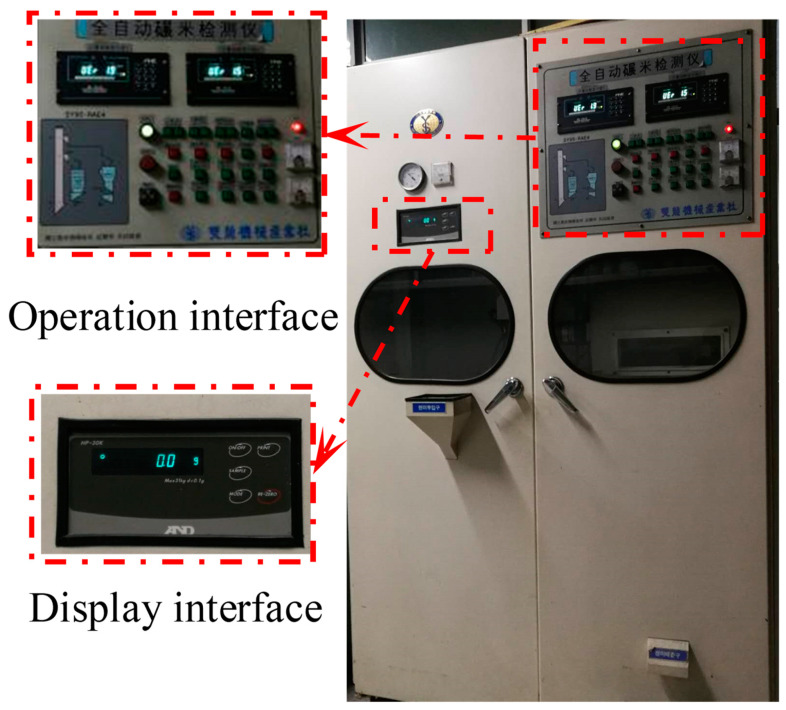
The vertical automatic rice mill system.

**Figure 3:**
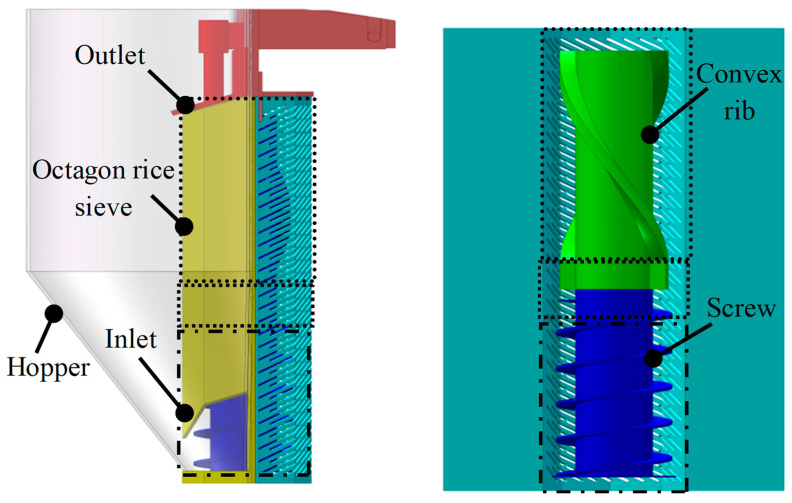
The schematic of the vertical rice mill.

The milling process used a vertical automatic rice mill system (SY95-PC+PAE5 type, Ssangyong Machinery Co., Korea), as shown in Figure 2. Milling is conducted under friction and the softer rice layers are removed by pressure and friction from the milling chamber or each other. The schematic of the vertical rice mill is shown in Figure 3. The down-drift weight of specimens can be displayed by a real-time monitor in the mill. Desired degrees of milling (DOMs) were obtained and calculated with the formula below [4]:

(1) $$DOM=\left(1-\frac{M_{a}}{M_{b}}\right)\times 100\%$$

where *M_a_* is weight of milled rice, g; *M_b_* is weight of initial BR, g.

**Figure 4:**
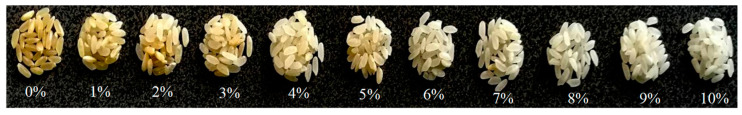
The milled rice samples subjected to different degrees of milling.

From the above, ten 200 g sub-samples were taken from polyethylene bags in the refrigerator and milled, varying between 1% and 10% DOM. Milling duration is recorded in Table 1. The milled samples (1~10%) and BR (0% DOM) are both shown in Figure 4.

### 2.3. Determination of Physical Properties

Physical properties of 0~10% DOM sub-samples were determined accurately according to the following methods, including length (*L*), width (*W*), thickness (*T*), aspect ratio (*R_a_*), volume (*V*), surface area (*S*), equivalent diameter (*D_p_*), sphericity (ϕ), bulk density (*ρ_b_*), true density (*ρ_t_*), porosity (ε), thousand kernel weight (*M_q_*), whiteness (*L_w_*), chromaticity (*B*), and coefficient of static friction (μ).

#### 2.3.1. Size and Shape

**Figure 5:**
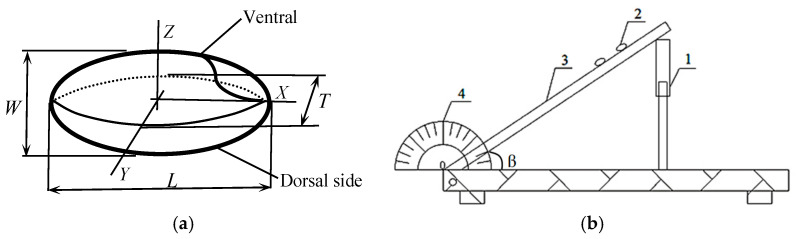
(**a**) A diagram of milled sample; (**b**) The apparatus to determine coefficient of static friction (1 screw nut, 2 kernels tested, 3 lifting table, 4 protractor).

The axial dimensions (Figure 5a), viz., length (*L*), width (*W*), and thickness (*T*), were measured using a digital vernier caliper (111N-102V-10 type, Guanglu, Nanjing, China) with an accuracy of 0.01 mm as principle dimensions. The 100 tested kernels were randomly selected from each DOM respectively. Other parameters of shape and size were then defined from these three linear dimensions.

The aspect ratio (*R_a_*) was determined from Equation (2) [33]:

(2) $$R_{a}=\frac{L}{W}$$

The volume (*V*) and surface area (*S*) of rice kernels were derived the following expressions [34], respectively:

(3) $$V=\frac{1}{4}[\left(\frac{\pi }{6}\right)L{\left(W+T\right)}^{2}]$$

(4) $$S=\frac{\pi {\left(WT\right)}^{1/2}L^{2}}{2L-{\left(WT\right)}^{1/2}}$$

The equivalent diameter (*D_p_*) is expressed as [34]:

(5) $$D_{p}={\left[L\frac{{\left(W+T\right)}^{2}}{4}\right]}^{1/3}$$

The sphericity (ϕ) was calculated through the following expression [34]:

(6) $$\phi =\frac{{\left(LWT\right)}^{1/3}}{L}$$

#### 2.3.2. Density and Weight

According to the different measuring methods of volume, the bulk density (*ρ_b_*) was the ratio of the mass (*W_b_*) of the grain to its total volume (*V_b_*), whereas the true density (*ρ_t_*) was the ratio of the mass (*W_t_*) of the grain to the solid volume (*V_t_*) occupied by the sample. The bulk density was determined by filling an empty 10 mL graduated cylinder with the rice samples, consolidated 3 times to achieve uniformity in the filling process as reported by Irtwange et al. [35], and the weight of the samples was obtained by subtracting the weight of the cylinder from the weight of the cylinder and samples.

The true density (*ρ_t_*) was measured by the toluene displacement method [34]. The true volume of the samples was measured by placing a known weight of samples in a measuring cylinder with toluene. The displaced volume was taken as the true volume of the sample. The process was replicated 5 times, and the bulk density and true density for each replication were calculated using the following expressions, respectively:

(7) $$\rho_{b}=\frac{W_{b}}{V_{b}}$$

(8) $$\rho_{t}=\frac{W_{t}}{V_{t}}$$

The porosity (ε) was calculated from bulk and true densities by using the following relationship [34]:

(9) $$\varepsilon =\left(\frac{\rho_{t}-\rho_{b}}{\rho_{t}}\right)\times 100\%$$

Thousand kernel weight (*M_q_*) was determined by the weight of 1000 randomly selected sound kernels by means of electronic balance (accuracy of 0.0001 g) and replicated five times for each DOM.

#### 2.3.3. Color and Lightness

The color of samples with different DOMs was measured using an automatic color meter (DC-P3 type, Xingguang, Beijing, China). The color meter adopts the CIE Lab *L*, *a*, *b* color space technique, where *L* indicates whiteness (*L_w_*) and *a* and *b* are the chromaticity coordinates. The whiteness (*L_w_*) can be measured and displayed directly on the automatic color meter. However, chromaticity (*B*) needs to be further calculated from the chromaticity coordinates (*a* and *b*) displayed on the automatic color meter. The measurement was replicated five times and the chromaticity (*B*) was calculated by the relation [36]:

(10) $$B=\sqrt{a^{2}+b^{2}}$$

#### 2.3.4. The coefficient of Static Friction

For measuring the static friction coefficient between the rice kernel and milling chamber wall, a device was designed through modification by Heidarbeigi et al. [33]. The apparatus consisted of several mechanisms, namely, a screw nut, protractor, and lifting table having the same material as that of the milling chamber wall (Figure 5b). The lifting table was raised gradually until the filled sample just started to slide down and the angle (β) between the lifting table and the horizontal surface was recorded. The process was replicated five times. The coefficient of static friction (μ) was calculated by the following formula:

(11) μ=tanβ

### 2.4. Statistical Analysis

We performed the multivariate analyses with the statistical package SPSS for Windows (version 23). All experimental data were expressed as mean ± standard deviations (SD) and subjected to a one-way analysis of variance (ANOVA). Significant difference was determined at the 0.05 probability level and post hoc tests were run by Tukey’s Test at a 5% probability level. HCA and PCA were also applied to the data set after standardization by the following formula:

(12) $$Z_{i}=\frac{X_{i}-\bar{X}}{\sigma_{\mathrm{sd}}}$$

where *Z_i_*—physical properties after standardization, *X_i_*—the measured physical properties, $\bar{X}$—the mean value of the measured physical properties, *σ_sd_*—the standard deviation of the measured physical properties.

### 2.5. Generalized Regression Neural Network (GRNN)

GRNN was developed as an alternative to the radial basis function neural network. It can be used for any regression problem in which an assumption of linearity is not justified [37]. In this study, nonlinear supervised models were investigated by using the GRNN for estimating different stages of rice milling. In general, the smoothness of the approximated function needs be controlled by the smooth factor, namely spread (σ), in the training phase of GRNN. Consequently, the optimal spread value in the GRNN model was selected by circularly measuring the Mean Square Error (MSE), as shown in Equation (13), between the predicted values and the experimental values to obtain the minimum in the training set using cross validation (cross validation 4 times). The performance of the GRNN models was evaluated at the correct rate.

(13) $$MSE=\sqrt{\frac{\sum_{i=1}^{n}{\left(y_{i}-{\hat{y}}_{i}\right)}^{2}}{n}}$$

## 3. Result and Discussion

### 3.1. Change in Physical Properties during Milling

As a result of the one-way analysis of variance from Table 1, the true density did not present significant differences, while the rest of the physical properties differed significantly during the milling. Similar investigations have been carried out to understand how physical properties of various rice cultivars influencing the DOM and similar results were found by Liu et al. [38]. However, the physical properties of the same cultivar of rice with different DOMs were not investigated in other studies.

In the case of size and shape, all the axial dimensions decreased significantly with an increasing DOM; *L*, *W,* and *T* declined 6.6%, 7.6%, and 14.1%, respectively, when the DOM reached 10%. Compared with *L* and *W*, *T* had more significant changes, suggesting that with the complex distribution of friction and rice attitude in the milling chamber, the brown rice was subjected to the most intense milling in the thickness direction [13]. Analogously, the equivalent diameter, volume, and surface area also showed a trend of decline. However, the aspect ratio and sphericity varied irregularly. Moreover, it must be noted that the standard deviations (Table 1) of all the dimensions at the beginning of the grinding were larger than that at 10% DOM, which might indicate that the BR was milled unevenly at the initial milling stage [39,40].

The thousand kernel weight decreased with the increase in DOM and varied from 21.01 to 18.25 g. The average weight of each grain was reduced by 13.8%, and the difference in weight was important for designing machine components such as those for ventilation and winnowers [34]. The kernels’ bulk density of varied from 761.27 to 824.45 kg/m^3^, and it rose with the increase in DOM; contrarily, porosity declined from 42.90 to 38.17% with the increase in DOM. Moreover, the true density practically did not present differences among the varieties in diverse DOMs. It can be inferred that the true density was not affected by milling, because the true density of rice might depend on the compact core endosperm instead of the removed bran layer. The variation tendency in these physical properties is consistent with those reported by Corrêa et al. [41]. In the case of the static coefficient of friction, the DOM increased gradually which led to the decrease in the static friction coefficient, due to the bran layer being clearly diminished. That may also explain why *L_w_* increased and *B* decreased with the increase in the DOM. The levels of pigments in the bran layer are significantly higher than the middle endosperm [7].

### 3.2. The Breakpoint Planning Method

**Figure 6:**
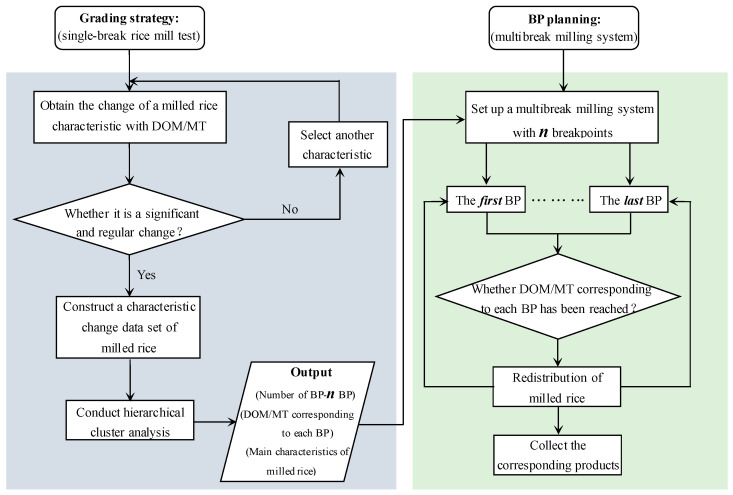
Breakpoint planning method illustrated with a flow diagram.

According to Tukey’s Test in Table 1, we can infer that each of the measured physical properties, except true density, aspect ratio, and sphericity, were regularly variable and significantly different (*p* < 0.05) between different DOMs. Therefore, we try to put forward a breakpoint planning method according to the changes of physical properties of milled rice during the whole rice milling process. The breakpoint planning method presented in this paper is divided into two stages, as illustrated in Figure 6. In the first stage, a continuous single-break rice milling experiment is required to obtain the characteristic changes of the milled rice during the whole milling process and to determine the grading strategy. Basically, this stage will produce the number of breakpoints (*n*) required the DOM or milling time (MT) corresponding to each breakpoint, and the representative characteristics of milled rice. In the second stage, using the grading strategy obtained in the first stage, a multibreak milling system with ***n*** breakpoints is constructed, and the actual breakpoint to which the milled rice obtained from each breakpoint belongs is estimated. Then, the milled rice obtained from each breakpoint is redistributed. Finally, milled rice products that meet different needs are obtained. The following sections describe each stage of the method in greater detail.

### 3.3. Grading Strategy

At present, the number of breakpoints of rice multibreak milling systems in rice mills is usually set by the experience of operators. In addition, the number of breakpoints in the rice multibreak milling system also needs to be adjusted according to the varieties of milled rice. However, to date, no objective method exists for planning the number of breakpoints in a reasonable way. Therefore, in order to propose a reasonable breakpoint planning method, it is necessary to grade the whole rice milling process to determine the number of breakpoints in the rice multibreak milling system. As shown in Figure 6, in the first stage, the characteristic changes of milled rice with DOM/MT are first analyzed. It is worth mentioning that, in this study, we mainly recorded and analyzed the characteristic changes of milled rice with DOM rather than MT. However, for the breakpoint planning method proposed in this study, it is also effective to record and analyze the characteristic changes of milled rice with MT. In addition, a further explanation of the changes in the characteristics of milled rice during the whole milling process has been given in Section 3. Therefore, in order to avoid subjective evaluation of the grade of milled rice only according to the characteristic changes of milled rice, we then introduce an objective grading method, that is, HCA.

#### Hierarchical Cluster Analysis

**Figure 7:**
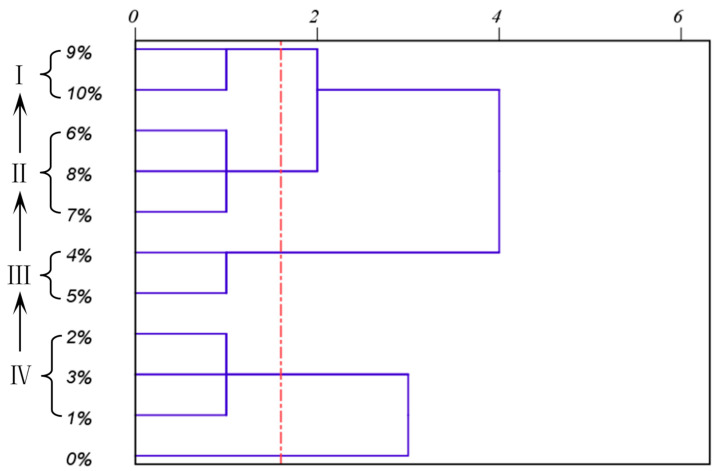
Dendrogram of HCA.

HCA is widely used to cluster objects into unknown groups and define new distinctive clusters [42]. In general, samples are grouped on the basis of similarities without taking into account the information about class membership, and the difference between observed individuals uses a defined metric (e.g., Euclidean, squared Euclidean, and Minkowski distance et al.) and clusters by Ward’s, median, and centroid clustering methods et al. In this work, Ward’s method and the squared Euclidean distance method were used as clustering methods. In order to avoid the effect of milling non-uniformity on the HCA, the data set for the HCA was made up of mean values of physical properties except for true density, aspect ratio, and sphericity. Then, standardization was conducted on the data set using Equation (12). The resultant information is presented in the dendrogram (Figure 7).

The BR was clearly separated from milled BR; therefore, it was a specific category. Then, 1~10% DOM was redefined as four grades: I, II, III, and IV. Group I contained a 9~10% DOM; group II contained a 6~8% DOM; group III contained a 4~5% DOM; and group IV contained a 1~3% DOM. These clusters might suggest that structure and composition of milled rice importantly affected physical properties during milling. Moreover, in respect of milling duration (Table 1), the average time was within 10 s and 20 s for DOMs of grades IV and III, respectively (removal fractions were designated as the bran layer and outer endosperm layer). However, for the DOM of grade II, milling duration lasted about 1 min and the milling speed was significantly reduced. It can be explained that compared to the bran layer and outer endosperm layer, the middle endosperm layer provides greater strength to hinder milling [15]. While it needs to take longer to achieve the DOM of grade I, the physical properties were almost unchanged (DOMs of 9% and 10% were clustered into one group firstly, due to the fact that they are the most similar, as seen in Figure 7).

### 3.4. Breakpoint Planning

#### 3.4.1. Setting up a Multibreak Milling System with n Breakpoints

In the first stage, the grading strategy can help operators classify the rice milling process objectively. According to the grading results of the first stage, a rice multibreak milling system with *n* (*n* is equal to the grading results obtained in the first stage; in this paper, *n* = 4) breakpoints can be constructed. However, the products of each single break are inevitably subjected to uneven milling [3], which undoubtedly reduces the effectiveness of the multibreak milling system. Therefore, on the basis of the grading strategy, the actual breakpoint which the milled rice obtains from each breakpoint needs be accurately assessed. Then, the milled rice obtained from each breakpoint needs be further redistributed. Further explanation of the redistribution strategy of milled rice is given below.

#### 3.4.2. Redistribution of Milled Rice

##### Properties Reduction

In order to further assess the actual breakpoint to which the milled rice obtained from each breakpoint belongs, we need to establish the relationship between the above gradually changing physical properties during the milling process and the four different stages obtained by HCA. However, at present, there are 12 properties that have been extracted. Those excessive properties increase computation time and storage memory, which may cause the assessment process to become more complicated and even decrease the performance of the assessment model. Therefore, a strategy is necessary to reduce the number of properties used in assessment. In this study, PCA was performed with the aim to better evaluate which physical properties were more significant in the characterization of the DOM. PCA was applied to measure 12 physical properties except the true density, aspect ratio, and sphericity. The three measured values of each physical property under different DOMs were chosen to form a 33 × 12 data set. Then, standardization was conducted on the data set using Equation (12). After standardization, all indices contribute equally to data set variance and carry equal weight in principal component calculation [29].

**Figure 8:**
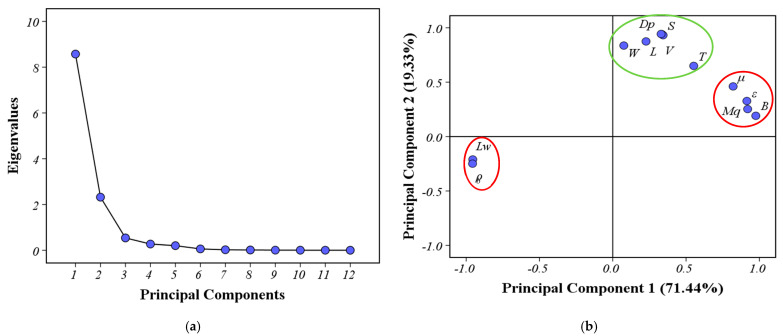
PCA plots. (**a**) The scree plot of principal components’ eigenvalues; (**b**) rotated loading plot for different factors on PC1 and PC2.

The scree plot (Figure 8a) shows eigenvalues of all principal components, in which the first two PCs with eigenvalues higher than 1 were extracted. Among the extracted PCs, PC1 was responsible for 71.44% of the total variance, while PC2 accounted for 19.33%. The first two principal components (PC1 and PC2) accounted for 90.77% of the total variance, which meets the general requirement of 85%, indicating that the two PCs, covering most of the information contained in 12 physical properties, might represent the changes of rice structure and composition characteristics from different aspects during milling. To further elucidate meaning of new comprehensive factors and discriminate between PC1 and PC2, the rotated solution of extracted PCs was calculated using the Varimax. The rotated loading plot (Figure 8b) shows that only the size and shape properties were co-located on the upper part of the rotated loading plot, indicating that they are the major constituents of the PC2. In addition, the PC1 consists mainly of other physical properties including *L_w_* and *ρ_b_* exhibiting high positive loadings, while *ε*, *μ*, *M_q_*, and *B* have the higher negative loadings. According to the results of the rotated loading, PC1 and PC2 can be clearly interpreted as an indirect evaluation factor (IEF) and direct evaluation factor (DEF) of the DOM, respectively. Additionally, PC1 and PC2 can be quantified through the following expression:

(14) $$PC1=a_{1}L+a_{2}W+a_{3}T+a_{4}V+a_{5}S+a_{6}D_{p}+a_{7}\rho +a_{8}\varepsilon +a_{9}\mu +a_{10}M_{q}+a_{11}L_{w}+a_{12}B$$

(15) $$PC2=b_{1}L+b_{2}W+b_{3}T+b_{4}V+b_{5}S+b_{6}D_{p}+b_{7}\rho +b_{8}\varepsilon +b_{9}\mu +b_{10}M_{q}+b_{11}L_{w}+b_{12}B$$

where $a_{1}$~$a_{12}$ and $b_{1}$~$b_{12}$ are the eigenvectors of PC1 and PC2, respectively. Corresponding eigenvectors are shown in Table 2.

**Figure 9:**
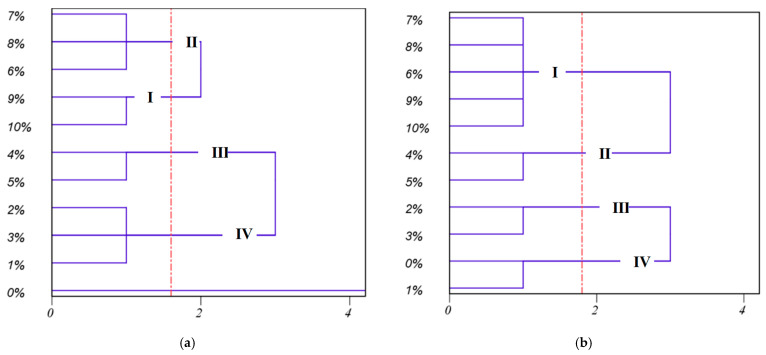
Dendrograms of HCA based on different physical properties. (**a**) Combination of physical properties including *V*, *S*, *D_p_*, *B*, and *L_w_*; (**b**) combination of physical properties including *B*, *L_w_*, *ρ_b_*, *ε*, and *M_q_*.

On the other hand, Table 2 also gives the rotated loadings, and, as expected, PC1 clearly discriminated *B*, *L_w_*, *ρ_b_*, *ε*, and *M_q_* from the other lower loadings of physical properties, while PC2 clearly discriminated *V*, *S*, and *D_p_* with higher loadings from the other physical properties. These results might suggest that a few representative physical properties can be selected from two comprehensive factors. Considering that PC1 represents the maximum variation of the data set, firstly, *B*, *L_w_*, *ρ_b_*, *ε*, and *M_q_* were selected as representative physical properties. However, two comprehensive factors (IEF and DEF) of the DOM might represent different changes in the DOM during rice milling. Therefore, *V*, *S*, and *D_p_* (from PC2) and *B* and *L_w_* (from PC1) were chosen as another group of representative physical properties. To validate whether the most representative variables were selected from the two groups of physical properties (*B*, *L_w_*, *ρ_b_*, *ε*, and *M_q_* and *V*, *S*, *D_p_*, *B*, and *L_w_*), the above HCA was conducted. The results obtained are shown as two dendrograms, respectively (Figure 9). The result (as shown in Figure 9b) of the HCA for *B*, *L_w_*, *ρ_b_*, *ε,* and *M_q_* was clearly distinct from the result of the original HCA (Figure 7), while for *V*, *S*, *D_p_*, *B,* and *L_w_*, the result (as shown in Figure 9a) of the HCA was essentially in agreement with that of the original HCA (Figure 7), except for clustering order (6%, 7%, and 8% DOMs are first clustered in a group in Figure 9a, while 9% and 10% DOMs are first clustered in a group in Figure 7). Therefore, these five physical properties (*V*, *S*, *D_p_*, *B*, *L_w_*) contained enough information to replace the original twelve physical properties in the evaluation of the DOM.

##### Assessment Model

The most important step in the redistribution strategy is to construct an assessment model to evaluate the actual breakpoint to which the milled rice belongs. However, the relationship between physical properties and DOM was nonlinear. In recent years, ANNs have been used in different fields of engineering because of their capability of extracting complex and nonlinear relationships [43]. In this study, the relationship model of physical properties and four grades (four breakpoints) was constructed by GRNN, which is one type of Radial Basis Function (RBF).

GRNN was applied to the data collection, which was consistent with that of PCA, and two-thirds of the data were used as training data and one-third of the data was used as test data. Both the inputs and output data were normalized to the range of [–1, 1] using Equation (16) [44].

(16) $$p_{n}=2\frac{p-p_{\min}}{p_{\max}-p_{\min}}-1$$

where *p_n_* is the normalized parameter, *p* denotes the actual parameter, *p_min_* represents a minimum of the actual parameters, and *p_max_* stands for a maximum of the actual parameters.

**Figure 10:**
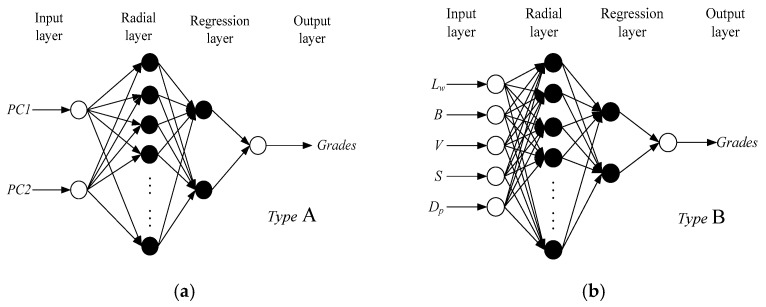
GRNN architectures with different input nodes. (**a**) Two input nodes: PC1 and PC2; (**b**) five input nodes: *V*, *S*, *D_p_*, *B*, and *L_w_*.

**Figure 11:**
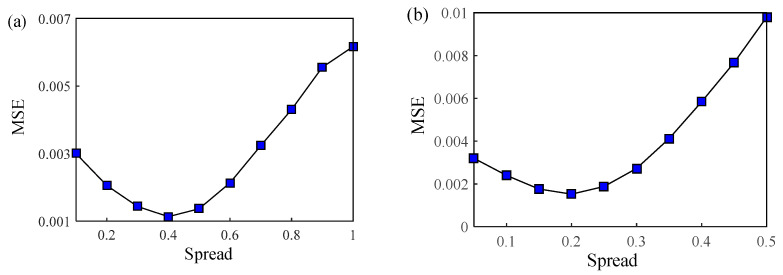
Changed MSE with different spread values of type A and type B in GRNN. (**a**) Type A: optimal spread is 0.4; (**b**) type B: optimal spread is 0.2.

According to the results of HCA and PCA, two GRNN architectures were constructed: type A has two input notes, which are PC1 and PC2 (PC1 and PC2 were calculated by Equations (14) and (15), respectively) (Figure 10a), while type B has five input notes that are *V*, *S*, *D_p_*, *B*, and *L_w_* (Figure 10b). The spread of type A was set from 0.1 to 1 at 0.1 intervals, and that of type B was set from 0.05 to 0.5 at 0.05 intervals. The changed MSE with the spread values of type A and type B are shown in Figure 11. The two curves both have a well-defined minima, with the optimal value of spread at 0.4 and 0.2. In this case, the optimal input and output of cross validation were also saved for constructing the optimal GRNN.

Four grades in the test data set (including 11 data points) were predicted using the optimal GRNN which was developed by the above training. The GRNN of type A showed the correct rate of 100%, while the GRNN of type B showed 90.91%. The predictive performance of type A was better than that of type B, suggesting that PC1 and PC2 do contain more information of physical properties to accurately assess results. However, considering the real-time evaluation of rice milling, type B is more likely to achieve success, due to the five input nodes (*V*, *S*, *D_p_*, *B*, *L_w_*) being easier to measure.

##### Effectiveness of Planning Method

To further verify the availability of the whole planning method including the grading strategy and redistribution strategy, the iso-color-phase differential staining (IDS) method was used as a standard method for determination of the processing degree of rice (Chinese standard GB 18105-2000, 2000) [45] and was compared with the estimated results obtained by the method proposed in this paper. First, the grading results proposed in this paper are the same as the standard grading method which divides the milled rice into four grades (Chinese standard GB 1354-2009, 2009) [46]. Then, to further verify the stability of the assessment model (GRNN model), the BR samples were milled to four different DOMs (this is an attempt to simulate the multibreak milling process). Next, we assessed the actual grades to which the milled rice obtained from each DOM belongs (the number of samples to be tested for each DOM is 40) and compared the results of the assessment with the grades of standard samples (note that we only use GRNN of type B). The results of the comparison are shown in Table 3. From our statistical results, if the multibreak planning method was not used, a large amount of rice would be overmilled or fail to reach the corresponding milling grades. In contrast, the planning method in this article can not only provide reasonable breakpoints but also more accurately redistribute the milled rice (the average correct rate can reach 94.28%, and grade II and grade III are more likely to be confused).

## 4. Conclusions

In this study, the variation of physical parameters during the rice milling process was analyzed using multivariate analysis. A breakpoint planning method was developed for the multibreak milling system based on the similarity of physical properties with the variation of DOM. Furthermore, a GRNN model was constructed to evaluate the actual breakpoints to which the milled rice obtained from each breakpoint belongs. The validation results show that the method has good accuracy. The main conclusions are as follows.

(1) The grading strategy based on the characteristic changes of the milled rice during the whole milling process enables the rice milling process to be divided into four different stages, group I contained a 9~10% DOM, group II contained a 6~8% DOM, group III contained a 4~5% DOM, and group IV contained a 1~3% DOM. The results of this stage, in a multibreak milling system, will help to initially determine the number of breakpoints required and DOM/MT corresponding to each breakpoint.

(2) The boundaries of each breakpoint are defined, which helps the operator to set the initial end point of each single breakpoint rice mill. For example, under the conditions of this study, the number of breakpoints of the multibreak milling system should be set to four and DOMs of 3%, 5%, 8%, and 10% can be used as the boundary of each breakpoint.

(3) The GRNN model is constructed to assess the actual breakpoints to which the milled rice obtained from each breakpoint belongs, because the products of each single break are inevitably subjected to uneven milling. The verification results show good accuracy (94.28%).

This work will help operators to plan the multibreak milling system of rice efficiently and objectively so as to significantly improve the commercial value of milled rice. Moreover, the method proposed in this study can also provide a reference for the breakpoint planning of the multibreak milling systems of other heterogeneous granular materials.

## Figures and Tables

**Table 1:** Physical properties of the milled rice subjected to different degrees of milling.

Physical Property	N	Brown Rice	Degree of Milling									
			1%	2%	3%	4%	5%	6%	7%	8%	9%	10%
Length (mm) **	100	5.932 ^a^	5.710 ^b^	5.708 ^b^	5.680 ^bc^	5.590 ^bcd^	5.700 ^b^	5.629 ^bcd^	5.509 ^d^	5.616 ^bcd^	5.552 ^cd^	5.542 ^d^
		(0.381)	(0.227)	(0.267)	(0.272)	(0.276)	(0.330)	(0.285)	(0.249)	(0.290)	(0.262)	(0.230)
Width (mm) **	100	2.398 ^a^	2.393 ^a^	2.392 ^a^	2.346 ^ab^	2.316 ^abc^	2.302 ^bcd^	2.286 ^bcde^	2.305 ^bcd^	2.257 ^cde^	2.229 ^de^	2.215 ^e^
		(0.394)	(0.119)	(0.122)	(0.123)	(0.108)	(0.131)	(0.130)	(0.323)	(0.109)	(0.102)	(0.100)
Thickness (mm) **	100	1.754 ^a^	1.692 ^ab^	1.670 ^b^	1.651 ^b^	1.640 ^bc^	1.584 ^cd^	1.568 ^abc^	1.533 ^de^	1.528 ^de^	1.527 ^de^	1.506 ^e^
		(0.075)	(0.413)	(0.076)	(0.087)	(0.077)	(0.082)	(0.084)	(0.088)	(0.069)	(0.082)	(0.070)
Aspect ratio **	100	2.509 ^b^	2.386 ^d^	2.386 ^d^	2.421 ^abcd^	2.414 ^acd^	2.476 ^abd^	2.463 ^abcd^	2.390 ^cd^	2.488 ^abd^	2.491 ^ab^	2.502 ^b^
		(0.291)	(0.134)	(0.140)	(0.160)	(0.168)	(0.195)	(0.175)	(0.190)	(0.186)	(0.170)	(0.160)
Volume (mm^3^) **	100	13.47 ^a^	12.47 ^ab^	12.32 ^bc^	11.87 ^bcd^	11.44 ^cde^	11.26 ^de^	10.94 ^def^	10.62 ^ef^	10.53 ^ef^	10.25 ^f^	10.04 ^f^
		(3.68)	(4.34)	(1.28)	(1.17)	(0.94)	(1.18)	(1.27)	(2.92)	(0.99)	(0.85)	(0.80)
Surface area (mm^2^) **	100	23.01 ^a^	21.89 ^b^	21.72 ^b^	21.22 ^bc^	20.70 ^cd^	20.52 ^cd^	20.12 ^de^	19.60 ^ef^	19.62 ^ef^	19.28 ^f^	19.02 ^f^
		(2.66)	(3.48)	(1.49)	(1.37)	(1.13)	(1.45)	(1.52)	(2.08)	(1.26)	(1.09)	(1.03)
Equivalent diameter (mm) **	100	2.94 ^a^	2.88 ^b^	2.87 ^b^	2.83 ^bc^	2.80 ^cd^	2.78 ^cde^	2.75 ^def^	2.73 ^efg^	2.72 ^fg^	2.70 ^g^	2.68 ^g^
		(0.20)	(0.21)	(0.10)	(0.09)	(0.08)	(0.1)	(0.11)	(0.17)	(0.08)	(0.07)	(0.07)
Sphericity **	100	0.492 ^ab^	0.499 ^a^	0.497 ^a^	0.493 ^a^	0.495 ^a^	0.482 ^bcd^	0.484 ^bcd^	0.488 ^abc^	0.478 ^cd^	0.480 ^cd^	0.477 ^d^
		(0.029)	(0.029)	(0.017)	(0.019)	(0.019)	(0.022)	(0.019)	(0.021)	(0.017)	(0.018)	(0.017)
Bulk density (kg/m^3^) **	5	761.27	779.10	783.69	784.25	810.16	812.28	814.35	819.52	822.39	822.47	824.45
		(1.96) ^f^	(0.92) ^e^	(2.97) ^de^	(3.99) ^d^	(2.05) ^c^	(0.06) ^c^	(1.07) ^bc^	(2.75) ^ab^	(0.25) ^a^	(1.20) ^a^	(0.28) ^a^
True density (kg/m ^3^) NS	5	1333.33	1333.34	1333.31	1333.33	1333.34	1333.30	1333.56	1333.21	1333.67	1333.32	1333.32
		(0.01) ^a^	(0.01) ^a^	(0.00) ^a^	(0.02) ^a^	(0.00) ^a^	(0.00) ^a^	(0.01) ^a^	(0.05) ^a^	(0.00) ^a^	(0.00) ^a^	(0.00) ^a^
Porosity (%) **	5	42.90 ^a^	41.57 ^b^	41.22 ^bc^	40.18 ^c^	39.24 ^d^	39.08 ^d^	38.92 ^de^	38.54 ^ef^	38.32 ^f^	38.31 ^f^	38.17 ^f^
		(0.15)	(0.07)	(0.22)	(0.30)	(0.15)	(0.00)	(0.08)	(0.21)	(0.02)	(0.09)	(0.02)
Static friction coefficient **	5	0.30 ^a^	0.29 ^a^	0.27 ^a^	0.27 ^a^	0.27 ^a^	0.26 ^a^	0.18 ^b^	0.18 ^b^	0.17 ^b^	0.16 ^b^	0.15 ^b^
		(0.01)	(0.01)	(0.01)	(0.00)	(0.01)	(0.02)	(0.04)	(0.00)	(0.02)	(0.00)	(0.01)
Thousand seed weight (g) **	5	21.01 ^a^	20.98 ^a^	20.30 ^b^	20.03 ^c^	19.94 ^c^	19.53 ^d^	19.14 ^e^	19.26 ^ef^	19.00 ^f^	18.40 ^g^	18.25 ^g^
		(0.07)	(0.05)	(0.03)	(0.03)	(0.01)	(0.09)	(0.04)	(0.06)	(0.08)	(0.13)	(0.02)
Whiteness (Lw) **	5	46.25 ^h^	47.90 ^g^	49.54 ^f^	50.11 ^ef^	51.15 ^de^	51.75 ^d^	52.93 ^c^	53.66 ^bc^	53.91 ^bc^	54.33 ^b^	55.86 ^a^
		(0.16)	(0.32)	(0.27)	(0.55)	(0.60)	(0.42)	(0.05)	(0.37)	(0.69)	(0.25)	(0.17)
Chromaticity (B) **	5	22.57 ^a^	19.84 ^b^	18.33 ^c^	17.76 ^c^	16.52 ^d^	15.92 ^d^	14.81 ^e^	14.13 ^ef^	13.47 ^fg^	13.00 ^g^	12.45 ^g^
		(0.41)	(0.41)	(0.24)	(0.64)	(0.33)	(0.25)	(0.13)	(0.42)	(0.14)	(0.32)	(0.40)
Time (s)		0	4	7	8	12	18	25	42	89	155	196

Notes: All data are presented as the mean with the standard deviation in parentheses. ** Is very significant (*p* < 0.01); ^NS^ Is statistically not significant (*p* > 0.05). The mean values in the same row followed by the same superscript letter denote that they are not significantly different (*p* > 0.05); in contrast, the different letter denotes that they are significantly different (*p* < 0.05).

**Table 2:** Physical properties of the milled rice subjected to different degrees of milling.

Physical Properties	Eigenvectors		The Rotated Loadings	
	PC1	PC2	PC1	PC2
Length (mm)	0.26	0.33	0.227	0.874
Width (mm)	0.21	0.38	0.075	0.837
Thickness (mm)	0.29	0.08	0.552	0.649
Volume (mm^3^)	0.30	0.32	0.336	0.940
Surface area (mm^2^)	0.30	0.31	0.343	0.932
Equivalent diameter (mm)	0.30	0.32	0.330	0.943
Bulk density (kg/m^3^)	−0.29	0.31	−0.955	−0.210
Porosity (%)	0.29	−0.28	0.920	0.253
Static friction coefficient	0.31	−0.13	0.821	0.461
Thousand seed weight (g)	0.31	−0.24	0.915	0.327
Whiteness (L_w_)	−0.30	0.29	−0.957	−0.249
Chromaticity (B)	0.29	−0.33	0.976	0.191

**Table 3:** The comparison between the assessment results and standard sample.

DOM	Number of Samples	Results of Assessment				Total Number of Each Grades		The Results Obtained by the Standard Method		Correct Rate
3%	40	I	II	III	IV	I (3%,5%,8%,10%)	44	I (3%,5%,8%,10%)	44	100%
		0	6	10	24					
5%	40	I	II	III	IV	II (3%,5%,8%,10%)	60	II (3%,5%,8%,10%)	57	92.86%
		3	19	18	0					
8%	40	I	II	III	IV	III (3%,5%,8%,10%)	32	III (3%,5%,8%,10%)	34	88.24%
		10	29	1	0					
10%	40	I	II	III	IV	IV (3%,5%,8%,10%)	24	IV (3%,5%,8%,10%)	25	96%
		31	6	3	0					
Total/average	160	44	60	32	24	160		160		94.28%

## Data Availability

All data are presented in this article in the form of figures and tables.
